# Self-Regulation of Learning and EFL Learners’ Hope and Joy: A Review of Literature

**DOI:** 10.3389/fpsyg.2022.833279

**Published:** 2022-02-17

**Authors:** Chenhan Huang

**Affiliations:** ^1^School of Public Affairs, University of Science and Technology of China, Hefei, China; ^2^School of Art, Anhui University of Finance and Economics, Bengbu, China

**Keywords:** self-regulation, positive psychology, motivation, joy, hope, emotional self-regulation

## Abstract

One of the new structures of positive psychology that has received special attention is academic self-regulation. It involves controlling one’s behavior, emotions, and thoughts to reach a long-term purpose. Learning how to self-regulate is an important skill that language learners learn both for emotional maturity and later social connections. There are various emotional factors that might be associated with the notion of self-regulation. Hope and joy are among the emotional factors that might influence language learners’ self-regulation; therefore, this literature review addresses the state of academic self-regulation in relationship with hope and joy. Reviewing the literature demonstrated that hope and joy affect language learners’ self-regulation. Hope and joy increase vitality, health, life satisfaction, and promote psychological wellbeing in EFL learners. They influence mental and physical health that is positively correlated with various scales, such as mental health, positive mood, avoidance of stressful life events, vitality and happiness in life, and problem-solving skill. The findings revealed that self-regulation in language learners depends on various demographic and educational factors that can improve learner’ language performance, mental health, and vitality of language learners. Curriculum designers and language teachers need to focus the findings of the current study on creating a more adequate and efficient language teaching environment.

## Introduction

During their educational period, language learners confront academic challenges, such as poor grades, academic stress, and reduced motivation (Fried, 2010). It is generally believed that appropriate strategies should be used and adapted to manage these challenges. Failure to address these challenges can cause mental health problems for language learners. Due to the high importance of promoting mental health in learning environments, this issue has received special attention from researchers in EFL educational contexts (Li et al., 2018). One of the concepts in contemporary academic and cognitive psychology is self-regulatory learning (Nakata, 2010).

Self-regulation plays an essential role in the processes and consequences of language learning and teaching (Grau and Whitebread, 2012; Wang and Guan, 2020). There are several definitions of self-regulated learning. Self-regulatory learning involves the strategies that language learners use to adjust their cognition. It is also the management strategies they use to control their learning process. According to Grau and Whitebread (2012), the most critical self-regulatory strategies are cognitive strategies, metacognitive strategies, and resource management strategies. Cognitive strategies refer to the strategies that language learners use to learn, recall, and comprehend. In other words, there are actions by which the language learners prepare new information to link and combine with previously known knowledge and store it in long-term memory. Metacognitive strategies monitor, control, and guide cognitive strategies. Language learners use metacognitive strategies to evaluate their understanding and determine how much time they need to study and overcome their challenges to reach an expected outcome. The third strategy of self-regulation notion is resource management strategies. It indicates that how much time they need to make optimal use of the allotted time. Language learners can control their life by managing time. Self-regulating individuals are aware of the impact of environmental factors on their accuracy when they are studying and can modify and change the situation to an appropriate one. In addition, Zimmerman (2011) proposed that self-regulation has three cyclical phases: the forethought phase, the performance phase, and the self-reflection phase.

Many studies have been conducted on the effect of emotion on learning. These studies emphasize that one-third of academic achievement is related to emotional issues (MacIntyre and Gregersen, 2012; Papi and Abdollahzadeh, 2012; Khalilzadeh and Khodi, 2018; Alavi et al., 2021); therefore, the relationship between emotion and self-regulatory learning must be identified. Pekrun (2006) classifies emotions into three categories: active and passive, positive and negative, powerful and action-based. Accordingly, hope as one of the factors that seems to be related to language learners’ academic self-regulation is an activating, positive emotion. According to Pekrun and Perry (2014), hope is one of the indicators of quality of academic life. It is experienced when achievement is positively evaluated, and the individuals have a sense of relative control over the situation. Another factor that seems to be related to language learners’ academic self-regulation is happiness or joy. Feeling happy has positive consequences for language learners’ lifestyles and academic achievement. This feeling increases the desire for behaviors that are related to academic success. Joy is a branch of positive psychology that has come to the attention of many psychologists in recent decades. They argue that both hope and joy might affect language learners’ self-regulation of learning.

In the past, many researchers have studied the relationship between cognitive and emotional processes with academic activity separately (Pekrun, 2009; Zimmerman, 2011; Sampson, 2012). However, today most psychologists pay attention to both cognition and emotion as integrated actions (Watkins et al., 2017; Goodwin et al., 2021; Pishghadam et al., 2021). Based on new theories, such as self-regulated learning, the components of cognition and emotion are considered as an intertwined and related set (Mellati et al., 2013; Seker, 2016; Derakhshan, 2021). Therefore, what should be considered in educational systems is the development and application of this new strategy as student-centered strategies in the student learning process (Dewaele et al., 2019). Nowadays, the adequacy of education with self-regulated learning strategies among language learners is one of the main challenges in higher education institutions. Despite the enormous psychological research on emotions, little of them has informed research on emotion, cognition, and self-regulatory learning strategies. Researchers know surprisingly little about the role of emotions in creating language learners’ academic self-regulation. Language researchers also know little about how language learners regulate their emotions, the relationship between learners’ emotions, such as hope and joy with learners’ academic self-regulation. In this article, the literature related to learners’ self-regulation is reviewed. The article begins with the empirical literature that focuses on hope and joy that language learners experience is summarized and critiqued. Next is an analysis of the various ways in which learners’ hope and joy may influence their academic self-regulation. Finally, several future directions for research are suggested.

### The Study

To cover different aspects of the topic, this literature review begins with a question, “What does the language teaching research say about language learners’ hope and joy and their academic self-regulation?” The objectives of this review are (a) to discuss the findings of studies that focus on this topic and (b) to provide important information to teachers, teacher educators, school principals, and policymakers on how to enhance teachers’ knowledge on improving learning opportunities and quality of education for English language learners. The following research question guided the study:

What does the educational research say about language learners’ hope and joy and their academic self-regulation?

## Review of Literature

Recently, the notion of positive psychology has received particular attention in general education and EFL contexts. Emotions and affective filters are among the most prominent elements of positive psychology (Magid and Chan, 2012; Mellati and Khademi, 2015, 2018; Khajavy and Ghonsooly, 2017; Wang et al., 2021). Hope and joy as subcategories of emotions directly relate to what EFL teachers feel in their teaching contexts. In general, emotions have many functions for human beings, and they serve service, survival, and adaptation. Some feelings are referred to as progressive emotions, that is, feelings that are directly related to the activities and consequences of progress (Dale, 2003; Boudreau et al., 2018; Gallagher and Lopez, 2018).

### Hope

In the Control-Value Theory of Pekrun Progress Emotions (2006), hope is presented as a positive, forward-looking emotion that motivates language learners to promote their language abilities. According to Chen and Park (2016), one of the most critical factors influencing motivation and academic achievement is rooted in the theory of hope. This theory is based on the positivist psychology approach as one of the new motivational models for educational research. According to Ong et al. (2006), hope is a process of thinking about a goal motivated to move toward that goal (agent) and the ways to reach it (the passage). Hope is a journey that requires three elements of destination (a goal), a road map (a course), and a vehicle (agent). The goals are the first factor for hope that is at the heart of the hope theory and can be short-term or long-term. Hence, hope is a dynamic cognitive-motivational system with many desirable effects in various fields, including language learning and teaching (Martin, 2014; Mellati et al., 2015b; Derakhshan et al., 2020). It means the ability to believe in feeling better in the future (Allen and Wright, 2014).

The results of research in this field show a positive relationship between the construct of hope and various variables, such as self-efficacy (Fathi and Derakhshan, 2019), academic performance (Chahkandi et al., 2016; Mellati and Khademi, 2020), life satisfaction (Csizer and Lukacs, 2010), academic success (Tsuda and Nakata, 2013), interest and motivation to continue education (Papi, 2018), intellectual conflict (Rose et al., 2018), general wellbeing (Greenier et al., 2021), and resilience (Ong et al., 2006), and its negative relationship with academic stress (Derakhshan et al., 2021), school violence (Dewaele and MacIntyre, 2014), and academic procrastination and self-disability (Dornyei, 2011). A study by Chew and Vanessa (2017) also showed that the overall expectation of 44% of the variance, according to Snyder’s theory of hope (Snyder and Lopez, 2007), is an active process in which people choose goals and try to achieve them by designing different paths. This basis seems to be related to the strategies that people adopt to achieve their educational goals. Hope is an emotion rooted in biological, psychological, and social resources and serves as a positive psychological stimulus in which individuals have a sense of the agent and the gateway to achieving their goals. It also, as a healing force, increases wellbeing (Csizer and Lukacs, 2010; Derakhshan et al., 2019).

Various studies (Dale, 2003; Ong et al., 2006; Martin, 2014; Chen and Park, 2016; Chew and Vanessa, 2017; Gallagher and Lopez, 2018; Mellati et al., 2018) have shown that hope is associated with positive emotions, feelings of self-worth, academic performance, and motivation in language learners. The results of these studies showed that students who have a higher level of hope are at a high level in terms of the orientation of achievement and education goals (Chen and Park, 2016). Language learners with high hopes report confidence and strength combined with a sense of self-worth, greater educational value, satisfaction, and lower levels of depression. Language learners with lower hopes report more depressive symptoms, fewer interpersonal relationships, and less ability to forget mistakes (Chew and Vanessa, 2017). They have less self-motivation and educational motivation than others (Ong et al., 2006). They also tend to avoid more or less effort in stressful situations (Gallagher and Lopez, 2018).

Early theories introduced hope as a one-dimensional structure that motivates language learners. Snyder considered it composed of willpower, navigation power, having a goal, and recognizing obstacles (Snyder and Lopez, 2007). Research has also shown that hope for positive emotion and a sense of self-worth are highly correlated and negatively associated with depression (Gallagher and Lopez, 2018). Hope has two components: cognitive (expectation of future events) and emotional (hope that these events are positive events and have desirable outcomes; Ong et al., 2006; MacIntyre and Gregersen, 2012; Mellati et al., 2015a). The emotional component of hope can predict the occurrence of positive events in the future and thus increase the chance of promoting healthy mental individuals (Dewaele and Alfawzan, 2018).

Research findings show that people with more hope showed higher self-esteem, better academic performance, and a more profound commitment to activities that led to better mental health (Chen and Park, 2016). Mental health is also known as one of the crucial dimensions of health and is one of the influential factors in the self-fulfillment of society. The World Health Organization defines mental health as the ability to communicate harmoniously and harmoniously with others, change and modify the individual and social environment, and resolve conflicts and personal inclinations in a logically and appropriately manner (Gallagher and Lopez, 2018). The importance of mental health in universities is valuable because it can be directly related to students’ academic achievement. Language learners’ life, unfamiliarity with the new educational environment, distance from family, lack of interest in the field of study, incompatibility with other people in the student living environment, and socio-economic problems can have a significant impact on their academic performance (Chew and Vanessa, 2017).

### Joy and Happiness

The second factor that seems to be related to language learners’ academic self-regulation is joy or happiness. Feeling happy has positive consequences for language learners’ lifestyles and academic achievement (Fowler and Christakis, 2008). This feeling increases the desire for behaviors that are related to academic success. Joy is a branch of positive psychology that has come to the attention of many psychologists in recent decades (Johnson, 2019). Happy learners have a calmer life, better general health, and a higher level of life satisfaction. Joy consists of three main components: positive emotion, life satisfaction, and lack of negative emotion. New studies in the field of joy show that being happy is learnable and the level of joy can be increased in a sustainable way (King, 2019). Lyubomirsky considers joy as the experience of feeling joy, satisfied, and happy, and that life is meaningful and valuable for a person (Lyubomirsky et al., 2005; Han, 2021).

Joy has three main components: positive emotion, life satisfaction, and lack of negative emotion, such as depression and anxiety (Fowler and Christakis, 2008). Positive relationships with others, purposeful life, personal growth, love of others, and nature are also components of joy. Joy has positive consequences on students’ lifestyles and academic success and increases their desire to engage in behaviors that are related to academic success. One of the characteristics of happy people is that they have high self-esteem and love themselves. These people pay a lot of attention to ethics and behave rationally (Meadows, 2014). The second characteristic is that happy people feel more personal control. Those who focus more on their abilities can cope with their stress. The third characteristic is that happy people are optimistic. These people are more successful and more comfortable than pessimists. The fourth characteristic is that happy people are extroverted and can communicate and collaborate with others (Saito et al., 2018). Happy people, whether alone or in the presence of others, feel satisfied and enjoy their lives and the lives of others (Seligman, 2011).

On the other hand, joy is one of the factors that has recently been discussed in the field of health psychology and is one of the most essential human psychological needs that has a significant impact on the formation of personality and mental health (Talebzadeh et al., 2019). Due to the importance of the subject, it is tried to provide more success in the life of the future group by increasing joy and vitality in the dormitory learners’ educational environment (Johnson, 2019). One of the activities that received particular attention in recent decades is happy methods to reduce the patients’ symptoms, especially depression and increase joy. Lyubomirsky Happiness is one of the most prestigious and popular collections. This treatment is less expensive and has a shorter treatment duration, and will bring about a faster recovery. In addition, it has no side effects compared to medication and has better safety (Lyubomirsky et al., 2005).

Numerous studies have shown that joy leads to engaging and productive activities. Joy as a positive emotion can facilitate interpersonal relationships, and its positive consequences increase the level of social action and health of language learners (Fowler and Christakis, 2008; Dewaele and MacIntyre, 2014; Meadows, 2014; Boudreau et al., 2018; Dewaele and Alfawzan, 2018; Teimouri, 2018; King, 2019). In recent years, psychologists and sociologists have taken a unique look at the experience of joy. Psychologists interested in the field of positive psychology have turned their attention to potential sources of positive emotions, such as feelings of joy (MacIntyre and Gregersen, 2012; Saito et al., 2018; Greenier et al., 2021; Pishghadam et al., 2021).

From a psychological point of view, there are two types of joy; a kind is obtained through tangible living conditions, such as marriage, education, job, and welfare facilities, which is called luxury joy (Allen and Wright, 2014). Another type of joy is influenced by inner states and personal perceptions, interpreted as mental joy (Boudreau et al., 2018). Joy is the material of energy, vitality, movement, and dynamism (Dewaele and Alfawzan, 2018). It is like a shield that protects a person against stress and problems and ensures his physical and mental health. Feelings of sadness, depression, and lack of joy can be affected by a person’s value places on himself. In some studies, academic and social problems due to self-worth become conditional, unstable, and fragile (Fowler and Christakis, 2008; Dewaele et al., 2019; Johnson, 2019).

### Self-Regulation

One of the strategies to promote hope and joy among language students is academic self-regulation, of which several definitions have been offered so far. According to Zimmerman (2011), academic self-regulation is a dynamic and constructive process in which learners choose goals for themselves and then try to control their cognition, behavior, and motivation following their intended purpose. Self-regulation is important in determining the activities that individuals pursue. It is also crucial in determining the amount of their effort and the distinct levels of their resistance to potential obstacles (Dornyei and Chan, 2013; Wang and Guan, 2020). Falout et al. (2009) use academic self-regulation in the form of three cognitive strategies that language learners use to learn and understand the material and subsequently recall their mental reserves. Metacognitive strategy means applying strategies, such as monitoring, evaluating, guiding, and modifying cognition to optimize cognition. Resource management strategies also indicate that the individuals use control methods to make their efforts more effective (Parker and Martin, 2009; Murphey and Falout, 2010; Fried, 2011).

Regarding the relationship between hope and academic self-regulation, Perry et al. (2008) believe that students with high education hope are more empowered to apply appropriate strategies to achieve their goals. The results of the related research also show the significant impact of positive emotions on metacognitive self-regulation, such as planning (Pishghadam et al., 2020), goal setting (Rose and Harbon, 2013), performance monitoring (Zimmerman, 2011), performance regulation (Frenzel et al., 2016), cognitive strategies (Derakhshan, 2021), metacognitive strategies (Fried, 2011), self-regulatory behaviors (Grau and Whitebread, 2012), and progress strategies (Li et al., 2018). In addition, the direct relationship between positive academic emotions and academic self-regulation and the direct relationship between negative academic emotions and academic self-regulation, and the significant impact of intellectual excitement of hope and joy on accepting academic help as one of the behavioral strategies of academic self-regulation have been reported in numerous studies (Rogat and Linnenbrink-Garcia, 2011; Rose et al., 2018; Fathi et al., 2021). Rogat and Linnenbrink-Garcia’s (2011) study on the relationship between self-regulation and learning strategies showed that self-regulation pays attention to language learners in managing learning strategies, including time management, seeking help, driving motivation to learn, monitoring learning success, and monitoring the effective use of effective learning strategies. In this regard, Bown (2009) researched the relationship between language learning and the application of self-regulatory strategies. He concluded that emotional factors are very effective in the application of these strategies.

Frenzel et al. (2016), in their research, showed that cognitive, motivational, and behavioral processes act as mediators between emotions and academic achievement. In addition, through influencing mediators, such as information processing (Csizer and Lukacs, 2010), selective attention (Dewaele et al., 2019), guidance (Dornyei and Chan, 2013), creating, and stopping self-regulation (Fathi et al., 2021), emotions also reduce and increase cognitive processing, such as problem-solving, memory and strategic thinking (Frenzel et al., 2016), increase controlled motivation and reduce automatic motivation (Fried, 2010), and increase self-regulatory behaviors (Goodwin et al., 2021). Therefore, it can be stated that they will have many positive educational effects (Khalilzadeh and Khodi, 2018).

The results of other studies indicate the positive effect of academic self-regulation on intrinsic motivation (Csizer and Lukacs, 2010), academic motivation (Dornyei and Chan, 2013), better academic performance (Falout et al., 2009), cognitive engagement (Fried, 2010), motivation and problem solving (Khalilzadeh and Khodi, 2018), academic adjustment (Magid and Chan, 2012), student satisfaction (Papi, 2018), and academic welfare (Pekrun, 2009), and also the negative effect of self-regulation on maladaptive behaviors and classroom maladaptation is in three dimensions: emotional, educational, and social level (Saito et al., 2018).

## Discussion

Reviewing the literature has shown that increasing hope in students is associated with reducing academic procrastination, better academic performance, improved wellbeing, joy, and adaptive behaviors, positive thinking, academic motivation and achievement motivation, optimism and vivacity, school success, and satisfaction.

The findings of the current study confirmed what Boudreau et al. (2018) found that there is a significant direct relationship between hope and joy and language learners’ academic performance. They also found a significant relationship between hope, self-efficacy, optimism, and academic achievement. Therefore, language learners who have more hope will be optimistic about their educational goals, feel more satisfied with their academic status, and as a result, might have more self-regulated than others.

Similar to Chew and Vanessa (2017), the findings of the present study asserted that language learners who have higher hopes use different paths to achieve their goals as an agent and seem to be more focused on issues and have less avoidance of confrontation than frustrated people. Furthermore, the findings revealed that language learners with higher levels of hope have more motivation and vitality to progress during their studies. In addition to increasing their knowledge, they consider the problems as a challenge that can be solved (Gallagher and Lopez, 2018). Regarding language learners’ academic self-regulation, the finding of the current study proved what Li et al. (2018) and Rose et al. (2018) showed in their studies that language learners with high academic self-regulatory ability achieve more academic success due to mastering the challenges. This has a positive effect on their educational vitality. In explaining this issue, it can be stated that people with high self-regulatory ability who are confident in their skills and deal with obstacles have higher academic vitality and therefore have higher academic achievement. In general, self-regulatory can predict academic achievement through self-regulation of movements and impulses (Perry et al., 2008; Zimmerman, 2011; Tsuda and Nakata, 2013; Frenzel et al., 2016).

Several studies emphasized the role of teachers in creating a friendly learning environment to enhance learners’ self-regulation. For instance, Allen and Wright (2014) stated that teachers can create an interactive and participatory atmosphere, engaging assignments, emphasize the importance of homework and other issues related to language learners’ educational environment to increase their academic self-regulation and vitality. Consistent with the findings of the present study, Fathi et al. (2021) found that language learners with high self-regulation have higher academic performance than language learners with low self-regulation. In other words, academic self-regulation predicts positive emotions. Grau and Whitebread (2012) also believe that self-confidence and self-regulation motivate students to engage in developmental tasks, unfavorable conditions, and future challenges. Overcoming challenges, such as a belief in personal abilities to deal with stressful environmental stimuli, is associated with adaptive functioning.

Similar to the findings of the present study, research in positive psychology has shown that hopeful and happy people have stronger social relationships with friends, spouses, neighbors, and relatives (Derakhshan et al., 2019), and hope increases vitality, health, increases life satisfaction, and promotes psychological wellbeing in individuals. Hope can increase positive emotions and reduce negative emotions. Hope is positively correlated with and predicts mental and physical health that is positively correlated with various scales, such as mental health, positive mood, avoidance of stressful life events, vitality and joy in life, and problem solving. In this regard, Martin (2014) showed that hope therapy increases the level of joy, meaning of life, and self-esteem and reduces anxiety and depression. Li et al. (2018) showed that self-regulation in language learners depends on various demographic and educational factors, which can improve the performance, mental health, and vitality of language learners.

Many studies have shown that people with high academic self-regulation feel more satisfied with their academic life and have more joy (Pekrun, 2006; Perry et al., 2008; Rose and Harbon, 2013; Papi, 2018; Rose et al., 2018). In line with what Dewaele and Alfawzan (2018) found the findings of the present study showed that there is a relationship between joy and academic vitality. Dewaele and MacIntyre (2014) found that language learners’ ability to reduce their helplessness and reduce stress, including joy, positive mood, and self-satisfaction, affects their success and academic motivation. In other words, joy in individuals leads to regulation and vitality, thinking power and academic achievement that is associated with quality of the life. Depression, on the other hand, reduces the power of reasoning, efficiency, and life expectancy during education.

The findings of Greenier et al. (2021) showed that there is a positive and significant relationship between language learners’ mental wellbeing and joy, and language learners who have higher wellbeing and joy in school and homework have less social anxiety and stress. On the other hand, people with low joy find normal daily events and test situations stressful. Furthermore, language learners with low joy consider normal daily events and exam situations as stressful and less lively and face many problems when faced with academic stressors. Therefore, to increase the joy of language learners, it seems that various programs, such as increasing sports activities and providing a lively atmosphere in school, would be helpful. These kinds of activities increase the spirit of vitality in them. Similarly, the results of King (2019) also showed that joy has a significant relationship with reducing anxiety, depression and diseases caused by academic stress, physical health, participation in group activities, feelings of altruism, and reducing the disease rate. Therefore, increasing joy can lead to mental wellbeing and health.

## Conclusion

The present review showed that joy and hope have mediating roles in the relationship with self-regulation. Although no research has been found that directly examines this mediation, given what has been said in explaining this finding, it can be said that people with lower hopes and self-regulation feel helpless and powerless in exercising control over life events when they are faced with obstacles. If their initial efforts to deal with problems are fruitless, they quickly give up hope and have less vitality during their studies. Similarly, the findings of Dornyei and Chan (2013) and Gallagher and Lopez (2018) studies demonstrated that the more hope language learners have, the more their progress and academic motivation. Language learners who have a higher level of hope are more focused on their goals and have greater academic joy and satisfaction. These people have perseverance, a cheerful spirit, and a serious determination to carry out activities because they believe that effort leads to their progress and satisfaction. Therefore, increasing hope in language learners increases motivation for progress and vitality in students. Thus, language learners’ beliefs and hopes about their abilities affect the performance of major academic activities, mental health and the reduction or increase of depression, academic stress, and the level of interest in intellectual activities and academic achievement.

Language learners’ hope for a bright future causes future efforts to overcome the challenges ahead. It can be said that hopeful and self-sufficient language learners have a happier spirit and are less prone to psychological problems and academic incompatibilities. Academic self-regulation and hope make the language learners belief that they can control academic tensions. In other words, language learners with high academic self-regulation are more hopeful about the future and plan for it, overcome challenges, and become more attuned to education once they reach equilibrium.

The pedagogical importance of these findings lies in the fact that language learners who perform successfully and operate in an adaptive learning environment have motivated knowledge that has repeatedly used cognitive and metacognitive strategies as opposed to language learners who performed poorly. They use less cognitive and metacognitive. That is, language learners who use self-regulated learning strategies have good motivational beliefs. In explaining this issue, it can be argued that the importance of self-regulation lies in meaningful learning; it allows learners to organize their knowledge in a coherent structure and integrate new information with previous structures. This strategy helps to integrate basic knowledge and new knowledge and helps to store information in language learners’ long-term memory through the connection between the learned materials.

Given that motivational beliefs play an important role in teaching and learning, it can be suggested that language teachers can focus on the mutual relationship between motivation and learning, so as interests and motivations develop, a language learner’s ability improves. Moreover, language teachers play a key role in improving learners’ self-regulation. According to the findings of the present study, the more hope and joy that teachers can create in language learners, the better their self-regulation and their learning achievements will be. This study can be duplicated in other educational contexts to shed light on latent aspects of culture and context in various learning environments. Future studies also can focus on the impact of other emotional factors, such as boredom, and fatigue on learners’ self-regulation.

## Data Availability Statement

The original contributions presented in the study are included in the article/supplementary material, and further inquiries can be directed to the corresponding author.

## Ethics Statement

The studies involving human participants were reviewed and approved by the University of Science and Technology of China Academic Ethics Committee. The patients/participants provided their written informed consent to participate in this study.

## Author Contributions

The author confirms being the sole contributor of this work and has approved it for publication.

## Conflict of Interest

The author declares that the research was conducted in the absence of any commercial or financial relationships that could be construed as a potential conflict of interest.

## Publisher’s Note

All claims expressed in this article are solely those of the authors and do not necessarily represent those of their affiliated organizations, or those of the publisher, the editors and the reviewers. Any product that may be evaluated in this article, or claim that may be made by its manufacturer, is not guaranteed or endorsed by the publisher.

## References

[ref1] Alavi S. M. Dashtestani R. Mellati M. (2021). Crisis and changes in learning behaviours: technology-enhanced assessment in language learning contexts. J. Further Higher Educ., 1–14. doi: 10.1080/0309877X.2021.1985977

[ref2] Allen J. M. Wright S. E. (2014). Integrating Theory and Practice in the Pre-Service Teacher Education Practicum. Teachers Teach. 20, 136–151. doi: 10.1080/13540602.2013.848568

[ref3] Boudreau C. MacIntyre P. D. Dewaele J. M. (2018). Enjoyment and anxiety in second language communication: an idiodynamic approach. Stud. Second Lan. Learn. Teach. 8, 149–170. doi: 10.14746/ssllt.2018.8.1.7

[ref4] Bown J. (2009). Self-regulatory strategies and agency in self-instructed language learning: a situated view. Modern Lan. J. 93, 570–583. doi: 10.1111/j.1540-4781.2009.00965.x

[ref5] Chahkandi F. Eslami Rasekh A. Tavakoli M. (2016). Efficacious EFL teachers’ goals and strategies for emotion management: the role of culture in focus. Iranian J. Appl. Ling. 19, 35–72. doi: 10.18869/acadpub.ijal.19.1.35

[ref6] Chen R. K. Park J. (2016). Positive psychology and hope as means to recovery from mental illness. J. Appl. Rehabil. Couns. 47, 34–42. doi: 10.1891/0047-2220.47.2.34

[ref7] Chew K. A. B. Vanessa A. (2017). Teaching from a place of hope in indigenous education. Anthropol. Newsl. 58, 265–269.

[ref8] Csizer K. Lukacs G. (2010). The comparative analysis of motivation, attitudes, and selves: the case of English and German in Hungary. System 38, 1–13. doi: 10.1016/j.system.2009.12.001

[ref9] Dale C. (2003). Sociology of hope. Humanit. Soc. 27, 293–294. doi: 10.1177/016059760302700310

[ref10] Derakhshan A. (2021). The predictability of Turkman students’ academic engagement through Persian language teachers’ non-verbal immediacy and credibility. J. Teach. Persian Speakers Lang. 10, 3–26. doi: 10.30479/jtpsol.2021.14654.1506t

[ref11] Derakhshan A. Coombe C. Zhaleh K. Tabatabaien M. (2020). Examining the roles of professional development needs and conceptions of research in English language teachers’ success. TESL-EJ 24, 1–28.

[ref12] Derakhshan A. Kruk M. Mehdizadeh M. Pawlak M. (2021). Boredom in online classes in the Iranian EFL context: sources and solutions. System 101:102556. doi: 10.1016/j.system.2021.102556

[ref13] Derakhshan A. Saeidi M. Beheshti F. (2019). The interplay between Iranian EFL teachers’ conceptions of intelligence, care, feedback, and students’ stroke. IUP J. Eng. Stud. 14, 81–98.

[ref14] Dewaele J. M. Alfawzan M. (2018). Does the effect of enjoyment outweigh that of anxiety in foreign language performance? Stud. Second Lang. Learni. Teach. 8, 21–45. doi: 10.14746/ssllt.2018.8.1.2

[ref15] Dewaele J. M. Chen X. Padilla A. M. Lake J. (2019). The flowering of positive psychology in foreign language teaching and acquisition research. Front. Psychol. 10, 21–28. doi: 10.3389/fpsyg.2019.02128, PMID: 31607981 PMC6769100

[ref16] Dewaele J. M. MacIntyre P. (2014). The two faces of Janus? Anxiety and enjoyment in the foreign language classroom. Stud. Second Lang. Learn. Teach. 4, 237–274. doi: 10.14746/ssllt.2014.4.2.5

[ref17] Dornyei Z. (2011). Researching complex dynamic systems: ‘retrodictive qualitative modeling’ in the language classroom. Lang. Teach. 47, 80–91. doi: 10.1017/S0261444811000516

[ref18] Dornyei Z. Chan L. (2013). Motivation and vision: an analysis of future L2 self images, sensory styles, and imagery capacity across two target languages. Lang. Learn. 63, 437–462. doi: 10.1111/lang.12005

[ref19] Falout J. Elwood J. Hood M. (2009). Demotivation: affective states and learning outcomes. System 37, 403–417. doi: 10.1016/j.system.2009.03.004

[ref20] Fathi J. Derakhshan A. (2019). Teacher self-efficacy and emotional regulation as predictors of teaching stress: an investigation of Iranian English language teachers. Teach. Eng. Lang. 13, 117–143. doi: 10.22132/TEL.2019.95883

[ref21] Fathi J. Greenier V. Derakhshan A. (2021). Teacher self-efficacy, reflection, and burnout among Iranian EFL teachers: the mediating role of emotion regulation. Iranian J. Lang. Teach. Res. 9, 13–37. doi: 10.30466/IJLTR.2021.121043

[ref22] Fowler J. Christakis N. (2008). Dynamic spread of happiness in a large social network: longitudinal analysis over 20 years in the Framingham heart study. Br. Med. J. 337, 2338–2347. doi: 10.1136/bmj.a2338, PMID: 19056788 PMC2600606

[ref23] Frenzel A. C. Pekrun R. Goetz T. Daniels L. M. Durksen T. L. Becker-Kurz B.. (2016). Measuring teachers’ enjoyment, anger, and anxiety: the teacher emotions scales (TES). Contemp. Educ. Psychol. 46, 148–163. doi: 10.1016/j.cedpsych.2016.05.003

[ref24] Fried L. (2010). Understanding and enhancing emotion and motivation regulation strategy use in the classroom. Int. J. Learn. 17, 115–130. doi: 10.18848/1447-9494/CGP/v17i06/47118

[ref25] Fried L. (2011). Teaching teachers about emotion regulation in the classroom. Australian J. Teach. Educ. 36, 1–11. doi: 10.14221/ajte.2011v36n3.1

[ref26] Gallagher M. W. Lopez S. J. (2018). The Oxford Handbook of Hope. New York: Oxford University Press.

[ref27] Goodwin A. P. Cho S.-J. Reynolds D. Silverman R. Nunn S. (2021). Explorations of classroom talk and links to reading achievement in upper elementary classrooms. J. Educ. Psychol. 113, 27–48. doi: 10.1037/edu0000462

[ref28] Grau V. Whitebread D. (2012). Self and social regulation of learning during collaborative activities in the classroom: the interplay of individual and group cognition. Learn. Instr. 22, 401–412. doi: 10.1016/j.learninstruc.2012.03.003

[ref29] Greenier V. Derakhshan A. Fathi J. (2021). Emotion regulation and psychological well-being in teacher work engagement: a case of British and Iranian English language teachers. System 97:102446. doi: 10.1016/j.system.2020.102446

[ref30] Han K. (2021). Fostering students’ autonomy and engagement in EFL classroom Through proximal classroom factors: autonomy-supportive behaviors and student-teacher relationships. Front. Psychol. 12:767079. doi: 10.3389/fpsyg.2021.767079, PMID: 34744946 PMC8564368

[ref31] Johnson M. K. (2019). Joy: a review of the literature and suggestions for future directions. J. Posit. Psychol. 15, 5–24. doi: 10.1080/17439760.2019.1685581

[ref32] Khajavy G. H. Ghonsooly B. (2017). Predictors of willingness to read in English: testing a model based on possible selves and self-confidence. J. Multiling. Multicult. Dev. 38, 871–885. doi: 10.1080/01434632.2017.1284853

[ref33] Khalilzadeh S. Khodi A. (2018). Teachers’ personality traits and students’ motivation: a structural equation modeling analysis. Curr. Psychol. 40, 1635–1650. doi: 10.1007/s12144-018-0064-8

[ref34] King P. E. (2019). Joy distinguished: teleological perspectives on joy as a virtue. J. Posit. Psychol. 15, 33–39. doi: 10.1080/17439760.2019.1685578

[ref35] Li C. Jiang G. Dewaele J. M. (2018). Understanding Chinese high school students’ foreign language enjoyment: validation of the Chinese version of the foreign language enjoyment scale. System 76, 183–196. doi: 10.1016/j.system.2018.06.004

[ref36] Lyubomirsky S. Sheldon K. M. Schkade D. (2005). Pursuing happiness: the architecture of sustainable change. Rev. Gen. Psychol. 9, 111–131. doi: 10.1037/1089-2680.9.2.111

[ref37] MacIntyre P. D. Gregersen T. (2012). Emotions that facilitate language learning: The positive-broadening power of the imagination. Stud. Second Lang. Learn. Teach. 2, 193–213. doi: 10.14746/ssllt.2012.2.2.4

[ref38] Magid M. Chan L. (2012). Motivating English learners by helping them visualize their ideal L2 self: lessons from two motivational programs. Innov. Lang. Learn. Teach. 6, 113–125. doi: 10.1080/17501229.2011.614693

[ref39] Martin A. M. (2014). How We Hope: A Moral Psychology. Princeton, NJ: Princeton University Press.

[ref40] Meadows C. M. (2014). A Psychological Perspective on Joy and Emotional Fulfillment. New York: Routledge.

[ref41] Mellati M. Fatemi M. A. Motallebzadeh K. (2013). The relationship between Iranian ELT instructors’ beliefs about language teaching and their practices in real classrooms. Engl. Lang. Teach. 6, 126–133. doi: 10.5539/elt.v6n4p126

[ref42] Mellati M. Khademi M. (2015). The impacts of distance interactivity on Learners' achievements in online Mobile language learning: social software and participatory learning. Int. J. Web-Based Learn. Teach. Technol. 10, 19–35. doi: 10.4018/ijwltt.2015070102

[ref43] Mellati M. Khademi M. (2018). Exploring teachers’ assessment literacy: impact on learners’ writing achievements and implications for teacher development. Australian. J. Teach. Educ. 43, 1–18. doi: 10.14221/ajte.2018v43n6.1

[ref44] Mellati M. Khademi M. (2020). MOOC-based educational program and interaction in distance education: long life mode of teaching. Interact. Learn. Environ. 28, 1022–1035. doi: 10.1080/10494820.2018.1553188

[ref45] Mellati M. Khademi M. Abolhassani M. (2018). Creative interaction in social networks: multi-synchronous language learning environments. Educ. Inf. Technol. 23, 2053–2071. doi: 10.1007/s10639-018-9703-9

[ref46] Mellati M. Khademi M. Shirzadeh A. (2015a). The relationships among sources of teacher pedagogical beliefs, teaching experiences, and student outcomes. Int. J. Appl. Ling. Eng. Lit. 4, 177–184. doi: 10.7575/aiac.ijalel.v.4n.2p.177

[ref47] Mellati M. Zangoei A. Khademi M. (2015b). Technology integration: EFL learners’ level of anxiety and their performance in writing tests. Int. J. Soc. Sci. Educ. 5, 240–252.

[ref48] Murphey T. Falout J. (2010). Critical participatory looping: dialogic member checking with whole classes. TESOL Q. 44, 811–821. doi: 10.5054/tq.2010.237337

[ref49] Nakata Y. (2010). Towards a framework for self-regulated language-learning. TESL Canada J. 27, 1–10. doi: 10.18806/tesl.v27i2.1047

[ref50] Ong A. D. Edwards L. M. Bergeman C. S. (2006). Hope as a source of resilience in later adulthood. Personal. Individ. Differ. 41, 1263–1273. doi: 10.1016/j.paid.2006.03.028

[ref51] Papi M. (2018). Motivation as quality: regulatory fit effects on incidental vocabulary learning. Stud. Second. Lang. Acquis. 40, 707–730. doi: 10.1017/S027226311700033X

[ref52] Papi M. Abdollahzadeh E. (2012). Teacher motivational practice, student motivation, and possible L2 selves: an examination in the Iranian EFL context. Lang. Learn. 62, 571–594. doi: 10.1111/j.1467-9922.2011.00632.x

[ref53] Parker P. D. Martin A. J. (2009). Coping and buoyancy in the workplace: understanding their effects on teachers’ work-related well-being and engagement. Teach. Teach. Educ. 25, 68–75. doi: 10.1016/j.tate.2008.06.009

[ref54] Pekrun R. (2006). The control-value theory of achievement emotions: assumptions, corollaries, and implications for educational research and practice. Educ. Psychol. Rev. 18, 315–341. doi: 10.1007/s10648-006-9029-9

[ref55] Pekrun R. (2009). “Global and local perspectives on human affect: implications of the control-value theory of achievement emotions,” in Contemporary Motivation Research: From Global to Local Perspectives. eds. Wosnitza M. Karabenick S. A. Efklides A. (Cambridge, MA: Hogrefe), 97–115.

[ref56] Pekrun R. Perry R. P. (2014). “Control-value theory of achievement emotions,” in International Handbook of Emotions in Education. eds. Pekrun R. Linnenbrink-Garcia L. (New York: Routledge), 120–141.

[ref57] Perry N. E. Hutchinson L. Thauberger C. (2008). Talking about teaching self-regulated learning: Scafflding student teachers’ development and use of practices that promote self-regulated learning. Int. J. Educ. Res. 47, 97–108. doi: 10.1016/j.ijer.2007.11.010

[ref58] Pishghadam R. Derakhshan A. Jajarmi H. Tabatabaee Farani S. Shayesteh S. (2021). Examining the role of teachers’ stroking behaviors in EFL learners’ active/passive motivation and teacher success. Front. Psychol. 12:707314. doi: 10.3389/fpsyg.2021.707314, PMID: 34354648 PMC8329251

[ref59] Pishghadam R. Ebrahimi S. Derakhshan A. (2020). Cultuling analysis: a new methodology for discovering cultural memes. Int. J. Soc. Culture Lang. 8, 17–34.

[ref60] Rogat T. K. Linnenbrink-Garcia L. (2011). Socially shared regulation in collaborative groups: an analysis of the interplay between quality of self-regulation and group processes. Cogn. Instr. 29, 375–415. doi: 10.1080/07370008.2011.607930

[ref61] Rose H. Briggs J. G. Boggs J. A. Sergio L. Ivanova-Slavianskaia N. (2018). A systematic review of language learner strategy in the face of self-regulation. System 72, 151–163. doi: 10.1016/j.system.2017.12.002

[ref62] Rose H. Harbon L. (2013). Self-regulation in second language learning: an investigation of the kanji-learning task. Foreign Lang. Ann. 46, 96–107. doi: 10.1111/flan.12011

[ref63] Saito K. Dewaele J. M. Abe M. In’nami Y. (2018). Motivation, emotion, learning experience and second language comprehensibility development in classroom settings. Lang. Learn. 68, 709–743. doi: 10.1111/lang.12297

[ref64] Sampson R. (2012). The language-learning self, self-enhancement activities, and self-perceptual change. Lang. Teach. Res. 16, 317–335. doi: 10.1177/1362168812436898

[ref65] Seker M. (2016). The use of self-regulation strategies by foreign language learners and its role in language achievement. Lang. Teach. Res. 20, 600–618. doi: 10.1177/1362168815578550

[ref66] Seligman M. E. P. (2011). Flourish: A Visionary New Understanding of Happiness and Well-Being. NY: Atria Books.

[ref67] Snyder C. R. Lopez S. J. (2007). Positive Psychology: The Scientific and Practical Explorations of Human Strengths. Thousand Oaks, CA, US: Sage Publications.

[ref68] Talebzadeh N. Elahi Shirvan M. Hassan Khajavy G. (2019). Dynamics and mechanisms of foreign language enjoyment contagion. Innov. Lang. Learn. Teach. 14, 399–420. doi: 10.1080/17501229.2019.1614184

[ref69] Teimouri Y. (2018). Differential roles of shame and guilt in L2 learning: how bad is bad? Mod. Lang. J. 102, 632–652. doi: 10.1111/modl.12511

[ref70] Tsuda A. Nakata Y. (2013). Exploring self-regulation in language learning: a study of Japanese high school EFL students. Innov. Lang. Learn. Teach. 7, 72–88. doi: 10.1080/17501229.2012.686500

[ref71] Wang Y. Derakhshan A. Zhang L. J. (2021). Researching and practicing positive psychology in second/foreign language learning and teaching: The past, current status and future directions. Front. Psychol. 12, 1–10. doi: 10.3389/fpsyg.2021.731721, PMID: 34489835 PMC8417049

[ref72] Wang Y. L. Guan H. F. (2020). Exploring demotivation factors of Chinese learners of English as a foreign language based on positive psychology. Revista Argentina de Clinica Psicologica 29, 851–861. doi: 10.24205/03276716.2020.116

[ref73] Watkins P. Emmons R. Greaves M. Bell J. (2017). Joy is a distinct positive emotion: assessment of joy and relationship to gratitude and well-being. J. Posit. Psychol. 13, 522–539. doi: 10.1080/17439760.2017.1414298

[ref74] Zimmerman B. (2011). “Motivational sources and outcomes of self-regulated learning and performance,” in Handbook of Self-Regulation of Learning and Performance. eds. Zimmerman B. Schunk D. (New York: Routledge), 49–64.

